# Lotus sign: The lumen-apposing metal stent that failed to bloom

**DOI:** 10.1055/a-2288-5076

**Published:** 2024-04-09

**Authors:** Gabriel Liu Yuan Cher, Yu-Ting Kuo, Chen-Ling Peng, Hsiu-Po Wang

**Affiliations:** 1 150819 Division of Gastroenterology, Department of General Medicine, Khoo Teck Puat Hospital, Singapore, Singapore; 2 38006 Division of Endoscopy, Department of Integrated Diagnostics & Therapeutics, National Taiwan University Hospital, Taipei, Taiwan; 3 38005 Department of Internal Medicine, National Taiwan University College of Medicine, Taipei, Taiwan; 4 38006 Department of Internal Medicine, National Taiwan University Hospital, Taipei, Taiwan

Endoscopic ultrasound-guided gastroenterostomy (EUS-GE) with the use of a lumen-apposing metal stent (LAMS) has emerged as a promising modality for the treatment of a malignant gastric outlet obstruction (MGOO) 1 . The design of single-step delivery systems of LAMSs can significantly reduce the risk of adverse events. However, the process of stent deployment is the crucial step in determining the success of EUS-GE. This report describes the case of a patient who underwent EUS-GE with the deployment of the proximal flange of the LAMS failing to open.

A 54-year-old man with advanced pancreatic adenocarcinoma presented with vomiting due to MGOO. After admission, EUS-GE with the wireless simplified technique 2 was performed using a 20-mm LAMS (Hot AXIOS stent; Boston Scientific, Marlborough, Massachusetts, USA). The distal flange of the LAMS deployed successfully. However, on deployment of the proximal portion, the most proximal end of the LAMS failed to open, resulting in a lotus-shaped flange in the stomach ( Fig. 1 , Video 1 ). Despite gentle manipulation of the internal sheath and allowing some time for the flange to expand, it still failed to bloom. A 20-mm extraction balloon catheter was passed through the narrow opening of the proximal end and inflated carefully to dilate the flange ( Fig. 2 , Video 1 ). This was followed by gushing of methylene-blue colored saline that had been previously irrigated into the jejunum. Fluoroscopy confirmed successful placement of the LAMS ( Fig. 3 ). A follow-up abdominal X-ray the next day revealed the stable position of the LAMS and the patient progressed uneventfully from a liquid to a regular diet. Caution should be taken in the event of a rare incomplete blooming of a LAMS flange, which indicates that the lumen-apposing force may be reduced and stent migration can occur. Early recognition and intervention may prevent stent misdeployment 3 .

**Fig. 1:**
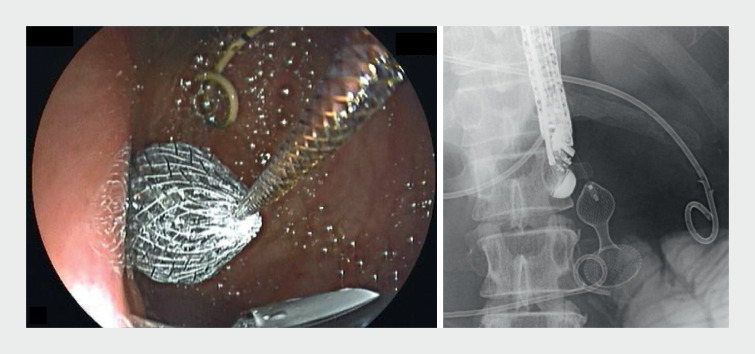
Endoscopy showed lotus sign of the proximal flange of the lumen-apposing metal stent (LAMS) resulting from the failure of the proximal end of LAMS to bloom.

**Fig. 2:**
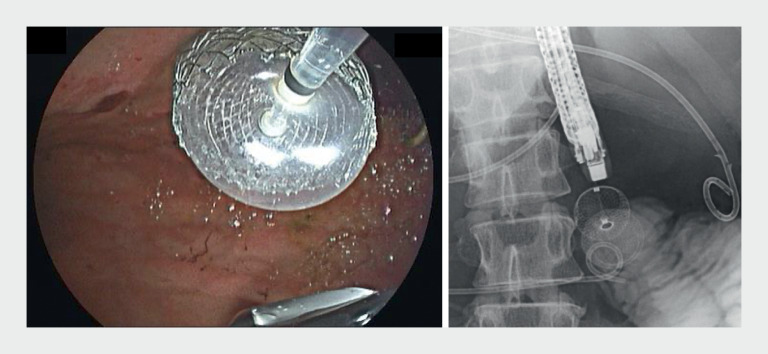
A 20-mm extraction balloon catheter was passed through the narrow opening of the proximal end of LAMS and inflated carefully to dilate the flange.

**Fig. 3:**
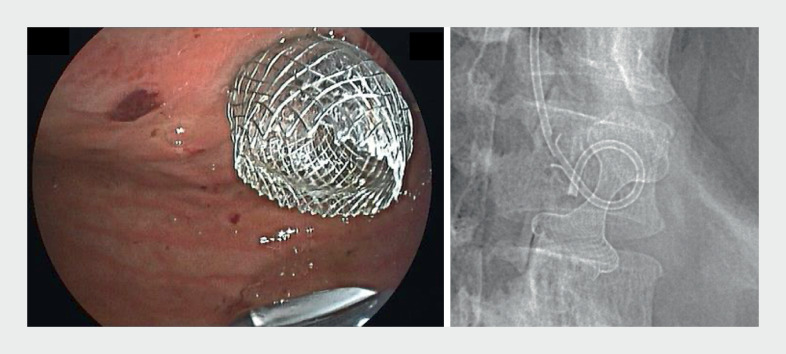
The proximal flange of LAMS satisfactorily opened after repeated balloon inflation.

Unexpected failure of the proximal flange of lumen-apposing metal stent to open and rescue management to prevent stent misdeployment.Video 1

Endoscopy_UCTN_Code_CPL_1AL_2AG

## Notes

Endoscopy E-Videos https://eref.thieme.de/e-videos
*E-Videos* is an open access online section of the journal *Endoscopy* , reporting on interesting cases and new techniques in gastroenterological endoscopy. All papers include a high-quality video and are published with a Creative Commons CC-BY license. Endoscopy E-Videos qualify for HINARI discounts and waivers and eligibility is automatically checked during the submission process. We grant 100% waivers to articles whose corresponding authors are based in Group A countries and 50% waivers to those who are based in Group B countries as classified by Research4Life (see: https://www.research4life.org/access/eligibility/ ). This section has its own submission website at https://mc.manuscriptcentral.com/e-videos .
